# Concordance and acceptability of a novel human papillomavirus self-collection tool for cervical cancer screening in economically developed regions: a prospective cohort study in Shanghai, China

**DOI:** 10.3389/fmed.2026.1760186

**Published:** 2026-03-05

**Authors:** Bowen Xu, Jingjing Liu, Hui Li, Tingting Zhang, Fang Li

**Affiliations:** 1 Department of Gynecology, Yueyang Hospital of Integrated Traditional Chinese and Western Medicine, Shanghai University of Traditional Chinese Medicine, Shanghai, China; 2 Department of Traditional Chinese Medicine, Shanghai East Hospital, Tongji University School of Medicine, Shanghai, China; 3 Department of Gynecology, Shanghai East Hospital, Tongji University School of Medicine, Shanghai, China

**Keywords:** cervical cancer screening, concordance, economically developed regions, HPV self- collection, patient- centered care, screening adherence

## Abstract

**Background:**

This study aimed to evaluate the concordance and clinical applicability of a novel cervical self-sampling device for human papillomavirus (HPV) genotyping, and to identify factors influencing women’s preference for self-collection in an economically developed setting.

**Methods:**

In this prospective paired-sample study conducted at Shanghai East Hospital (September 2024–February 2025), 276 eligible women from gynecological outpatient clinics underwent sequential cervical sampling: self-collection followed by clinician-collected sampling. Both specimens were analyzed in parallel for HPV genotyping using standardized protocols. Using clinician sampling as the reference standard, concordance parameters including sensitivity, specificity, and predictive values were calculated with 95% confidence intervals (CIs). Patient-reported outcomes (*n* = 268 valid responses) on acceptability, comfort, and preference were collected through a structured questionnaire.

**Results:**

The self-sampling method showed excellent concordance with clinician sampling (*κ* = 0.937; 95% CI:0.89–0.98). Concordance with clinician sampling was as follows: sensitivity 91.4% (95% CI: 83.2–96.5%), specificity 100% (95% CI: 98.2–100%), accuracy 97.5% (95% CI: 94.8–99.0%), positive predictive value 100% (95% CI: 95.0–100%), and negative predictive value 96.5% (95% CI: 93.2–98.5%). While 41.4% of participants initially preferred self-sampling, this proportion increased to 56.7% when cost considerations were introduced. Key determinants for self-sampling preference included: comfort of home-based collection (59.2%), perceived reduction in pain through self-controlled sampling pressure (57.2%), and privacy preservation (56.6%). Multivariate analysis revealed significant associations between sampling preference and age (*p* < 0.0001), HPV vaccination status (*p* = 0.0064), previous screening experience (*p* = 0.0064), and comfort perception (*p* < 0.0001).

**Conclusion:**

The novel self-sampling device achieves high concordance comparable to clinician-collected specimens while demonstrating superior patient acceptability. Its advantages in privacy protection, procedural comfort, and cost-effectiveness position it as a promising complementary strategy to enhance cervical cancer screening coverage, particularly among under-screened high-risk populations.

## Introduction

Cervical cancer represents a significant global threat to women’s health, ranking as the fourth most common malignancy and the fourth leading cause of cancer-related mortality among women worldwide (1). The World Health Organization (WHO) estimates approximately 604,000 new cases and 349,000 deaths globally in 2022, with cervical cancer being the most frequently diagnosed cancer among women in 25 countries (2). In China, national cancer registry data for 2022 reported 150,700 new cervical cancer cases among a total of 4,824,700 new cancer diagnoses (3). Persistent infection with high-risk human papillomavirus (HR-HPV) genotypes, particularly HPV 16 and 18, is the primary etiological factor in cervical carcinogenesis. These oncogenic variants disrupt the regulatory mechanisms of the cervical epithelial cell cycle, ultimately leading to malignant transformation (4–6). Notably, HPV 16 and 18 are responsible for approximately 70% of cervical cancer cases globally. Regional data from Henan Province, China, indicates detection rates of HPV 16, 58, and 52 as high as 75.7% among cervical cancer patients (7). The disproportionately higher mortality rates observed in developing countries, largely attributable to limited screening resources, underscore the critical importance of HPV-based screening in cervical cancer prevention (8).

HPV genotyping has become a cornerstone of cervical cancer screening, offering superior sensitivity and negative predictive value compared to conventional cytological methods such as the Pap smear (9). This molecular approach enables earlier detection of high-risk HPV infections, facilitating timely interventions that can reduce cervical cancer incidence and mortality. Furthermore, HPV genotyping provides essential information for risk stratification through the identification of specific high-risk genotypes, supporting personalized clinical management and surveillance protocols (10). In January 2023, China’s National Health Commission launched the Accelerating the Elimination of Cervical Cancer Action Plan (2023–2030), mandating nationwide improvements in cervical cancer prevention systems and comprehensive treatment capabilities. Concurrently, the Chinese Guidelines for Cervical Cancer Screening (I) (2023) established HR-HPV nucleic acid testing as the primary screening modality (11). Despite these advancements, the implementation of HR-HPV screening in China remains suboptimal, with significant regional disparities in coverage rates. These challenges stem primarily from limited healthcare accessibility and variable patient acceptance of screening procedures.

Self-sampling for diagnostic testing has gained significant global traction, particularly during the COVID-19 pandemic (12). Conventional cervical screening methods, which rely on clinician-collected Pap smears or HPV tests, face limitations due to disparities in healthcare resources and women’s concerns regarding privacy and procedural discomfort. These factors contribute to persistently low screening coverage in underserved regions (13). The clinical utility of HPV self-sampling was first demonstrated in 1999 by Hillemanns et al., who reported a sensitivity of 93% for detecting cervical intraepithelial neoplasia grade 2 or worse (CIN2+) (14). Global implementation studies confirm that HPV self-sampling significantly enhances screening accessibility through decentralized specimen collection. Systematic reviews demonstrate that home-based self-sampling programs achieve screening participation rates several times higher than traditional clinic-based approaches (15, 16). This strategy has proven particularly effective among hard-to-reach populations. For example, in Canada’s Indigenous communities, its convenience and privacy advantages have helped overcome cultural barriers to screening participation (17). Economic analyses further highlight the cost-effectiveness of self-sampling, prompting large-scale validation studies across low- and middle-income countries (LMICs) in the Americas, Asia, and Africa (18–22). Recognizing its transformative potential, the World Health Organization (WHO) now endorses self-sampling as a cornerstone strategy for eliminating cervical cancer in LMICs (23).

Despite this progress, global adoption remains limited. A 2023 landscape analysis revealed that only 35% of 139 countries with formal screening guidelines promote HPV primary screening. Of these, merely 17 countries (representing 35% of HPV-adopting nations) officially recommend self-sampling (24). In China, persistent challenges include suboptimal HR-HPV screening rates and pronounced regional disparities in implementation. Integrating self-sampling for HR-HPV nucleic acid detection could address these gaps by leveraging its operational simplicity and privacy protection features, thereby expanding screening coverage and advancing national cervical cancer prevention objectives. We are particularly interested in understanding whether eligible screening-age women in economically developed regions of China, such as Shanghai—where medical infrastructure is advanced—exhibit the same level of acceptance for HPV self-sampling as women in low- and middle-income regions. Thus, this study was performed to evaluate the concordance of a novel cervical self-sampling device with clinician-collected specimens for HR-HPV detection and to assess its acceptability. Moreover, the patient acceptance of self-sampling technology and identify the demographic, socioeconomic, and cultural determinants that influence screening participation rates, thereby generating evidence for protocol optimization were also evaluated.

## Materials and methods

### Study design

This prospective paired-sample study employed a cross-sectional comparative design to evaluate the diagnostic validity of a cervical self-sampling device for HPV genotyping. Participants performed self-collected vaginal sampling followed immediately by physician-obtained cervical specimens. This sequential protocol eliminated potential confounding effects from prior clinical procedures on cytological integrity. We quantified the agreement between the two sampling methods for HPV detection by calculating sensitivity, specificity, and concordance. We explicitly clarify that the clinician sample served as the comparator for the molecular test, not as a reference standard for histopathological diagnosis. A structured questionnaire systematically assessed participants’ acceptance of the self-sampling technology, their perceived comfort levels during the procedure, and their methodological preferences between the self-administered and clinician-based approaches.

### Study site and participants

The study was conducted at the Cervical Specialty Clinic of the Department of Gynecology, Shanghai East Hospital, between September 1, 2024, and February 28, 2025. Eligible women attending the gynecology outpatient clinic were recruited consecutively. The inclusion criteria were: (1) aged 25–65 years; (2) sexually active; (3) willing to provide written informed consent. The exclusion criteria included: (1) sexual intercourse within 24 h preceding specimen collection; (2) vaginal douching or use of intravaginal medication within 3 days prior to sampling; (3) application of acetic acid or iodine solutions prior to sampling; (4) current use of oral contraceptives or vaginal medications; (5) pregnancy or within 6 weeks postpartum; (6) history of cervical resection or total hysterectomy; and (7) declined study participation or inability to complete study procedures.

### Sample size estimation

The sample size was calculated based on an anticipated concordance (*κ* = 0.95) with a 95% confidence interval width of ±0.05, derived from prior research, and an expected HPV infection rate of 30%. This yielded a required sample of 250 cases. Accounting for an estimated 10% attrition rate, the final recruitment target was set at 275 participants.

### Study procedures

**Figure 1:**
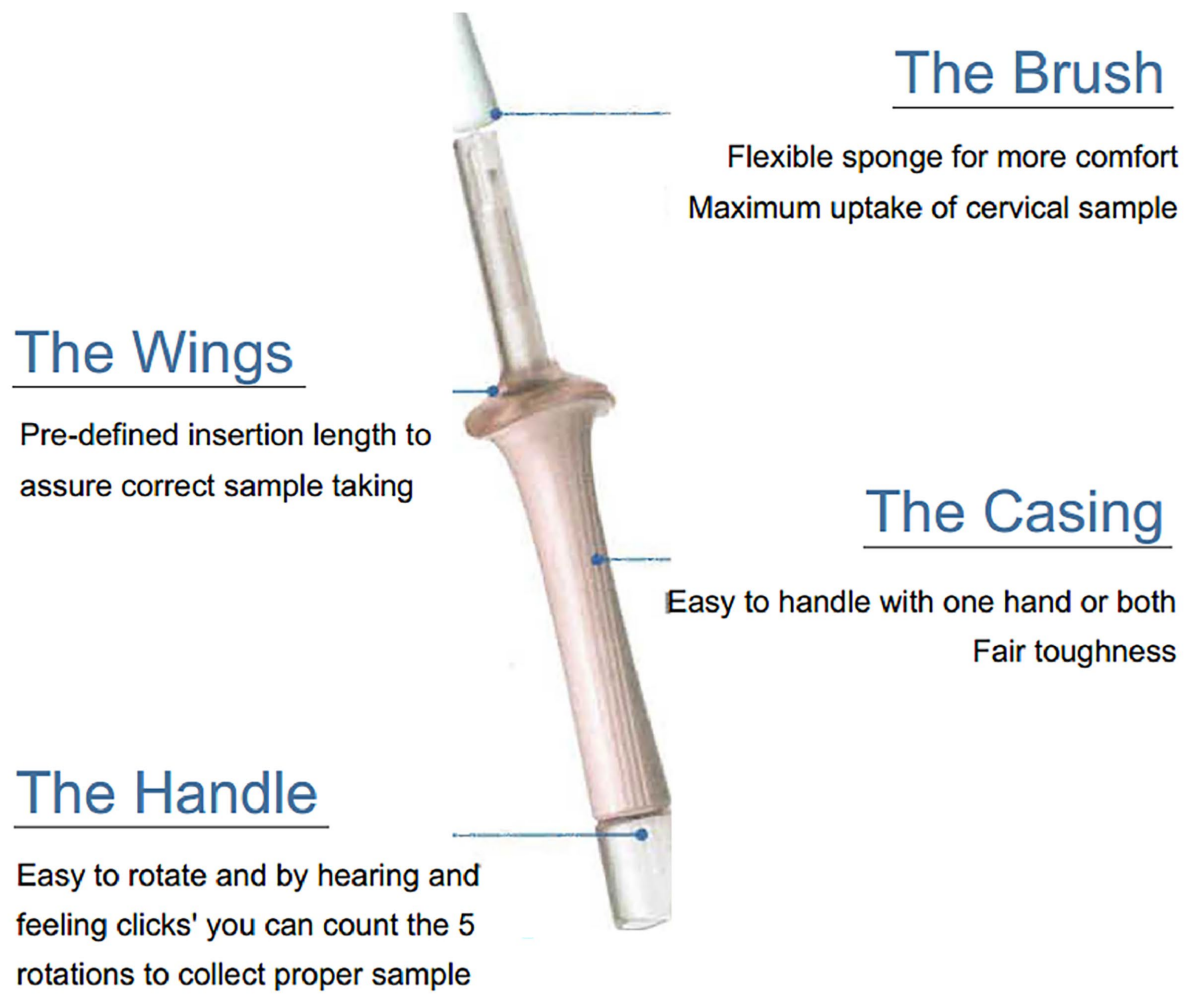
Design and key features of the novel cervical self-sampling device.

Certified research nurses at the clinic explained the study protocol and obtained written informed consent from eligible participants. Research assistants then provided verbal instructions on operating the self-sampling device and placed illustrated instructions in a dedicated sampling area. Participants performed self-sampling independently using the provided materials. Subsequently, attending gynecologists (with ≥5 years of experience) collected cervical specimens using the clinician-administered method. After specimen collection, all participants completed a standardized questionnaire evaluating both sampling methods (Figure 1).

### Sampling methods

#### Self-sampling device

The study utilized a sterile, single-use cervical self-sampler (Pinjia Health Technology (Hunan) Co., Ltd.; Batch No.: Xiangmei Note Quarantine 20,232,181,191; Manufacture date: September 2023). Its key design features include: (1) Ergonomic structure: comprising a handle, a telescoping shaft (14 ± 0.5 cm in length), and a sampling head (1.5 cm in diameter); (2) 360° rotating sampling head: ensures complete contact with the cervix for adequate cellular material collection; (3) Soft silicone material: minimizes discomfort during insertion; and (4) Anatomical compatibility: dimensions are optimized for the vaginal anatomy of Chinese women. The device holds a Chinese utility model patent and a design patent (Patent Nos. ZL 2022 2153373.5 and ZL 2023 0862725.7). A visual representation of the device is provided in Figure 1.

#### Self-sampling procedure

Participants performed self-sampling in a private space, following the product instructions: (1) wash hands and open the product package; (2) assume a comfortable position (standing or semi-squatting). Hold the sampler handle with one hand and gently spread the vulva with the other; (3) gently insert the sampler into the vagina until slight resistance is felt (typically at a depth of approximately 14 cm); (4) press the button on the sampler handle to release the tip. Rotate the grip 5–8 times (approximately 360° rotation) to ensure full contact with the cervical surface; (5) retract the tip and gently withdraw the sampler; and (6) place the sampling head into the supplied cell preservation solution, detach it by breaking or twisting, and seal the preservation tube. The entire process typically took 3–5 min. A research assistant remained on standby nearby but did not intervene during the procedure.

#### Clinician sampling procedure

Approximately 5 min after self-sampling, an attending gynecologist collected a cervical specimen using a standard exfoliative cell collection method. The participant was placed in the lithotomy position. After inserting a speculum to visualize the cervix, the clinician used a sampler of the same type to collect cells from the cervical surface and canal. The sampling head was then placed into a labeled cytological preservation tube.

### Sample processing and HPV detection

Both self-collected and clinician-collected specimens were preserved in cytological preservation solution (Cida Biotechnology, Guangzhou; Batch No. GZ20230812) at 2–8 °C for 24–48 h. Specimens were subsequently transported at ambient temperature (15–25 °C) to Zhengzhou Aiwidi Medical Laboratory, an independent third-party laboratory, with a transit time not exceeding 72 h.

HPV genotyping was performed using a PCR-reverse dot hybridization assay (Human Papillomavirus Genotyping Kit, Type 23; Yaneng Biotechnology, Shenzhen; Batch No. SZ20230915; Registration No. 20173400219). For the primary analysis, a sample testing positive for any of the 23 HPV types (6, 11, 16, 18, 31, 33, 35, 39, 42, 43, 44, 45, 51, 52, 53, 56, 58, 59, 66, 68, 73, 81, 82, 83) was considered ‘HPV positive.’ A secondary analysis was conducted considering only the 18 high-risk (HR) HPV types (16, 18, 31, 33, 35, 39, 45, 51, 52, 53, 56, 58, 59, 66, 68, 73, 82, 83) as clinically relevant positives. All specimens were processed within 7 days of laboratory receipt. To minimize inter-batch variability, specimens from the same shipment were analyzed concurrently.

### Questionnaire administration

After completing both sampling methods, participants filled out a structured questionnaire covering five domains: (1) demographics: age, educational attainment, HPV vaccination history, and cervical screening history; (2) procedural experience: evaluated on 10-point scales for each method (comfort level: 1 = least satisfied to 10 = most satisfied; pain intensity: 0 = no pain to 10 = worst pain imaginable); (3) sampling preference: choice between self-sampling, clinician-sampling, or no preference, with selection of primary rationale (privacy, comfort, professional expertise); (4) self-sampling implementation assessment: questions regarding operational concerns and perceived need for assistance; and (5) cost–benefit analysis: comparison of method preferences under different pricing scenarios (approximately CNY 200 for self-sampling vs. CNY 300 for clinician-sampling).

### Statistical analysis

Statistical analyses followed the per-protocol principle, including only subjects with complete paired HPV test results. Three analytical frameworks were employed: (1) concordance evaluation: kappa statistics (with 95% CI) quantified inter-method agreement for HPV detection. Interpretation thresholds were: >0.8 = very good; 0.61–0.80 = good; 0.41–0.60 = moderate; 0.21–0.40 = fair; <0.21 = slight; (2) concordance evaluation: sensitivity, specificity, accuracy, positive predictive value (PPV), and negative predictive value (NPV), and their 95% CIs were calculated for self-sampling compared to clinician sampling. We note that predictive values (PPV, NPV) are reported but their interpretation is limited in the absence of disease prevalence (CIN2+) data. McNemar’s test was used to compare paired proportions; (3) acceptability profiling: Categorical variables were presented as frequencies (percentages), and continuous variables as mean ± standard deviation or median (interquartile range). Demographic predictors were assessed using χ^2^ tests, with association strength measured by Cramér’s V (0.1–0.3 = weak; 0.3–0.5 = moderate; >0.5 = strong). Determinants of self-sampling acceptability were identified through multivariable logistic regression. All analyses were performed using SPSS 25.0 (IBM Corp.), with a two-sided *p*-value < 0.05 considered statistically significant.

## Results

### Study population characteristics

**Figure 2:**
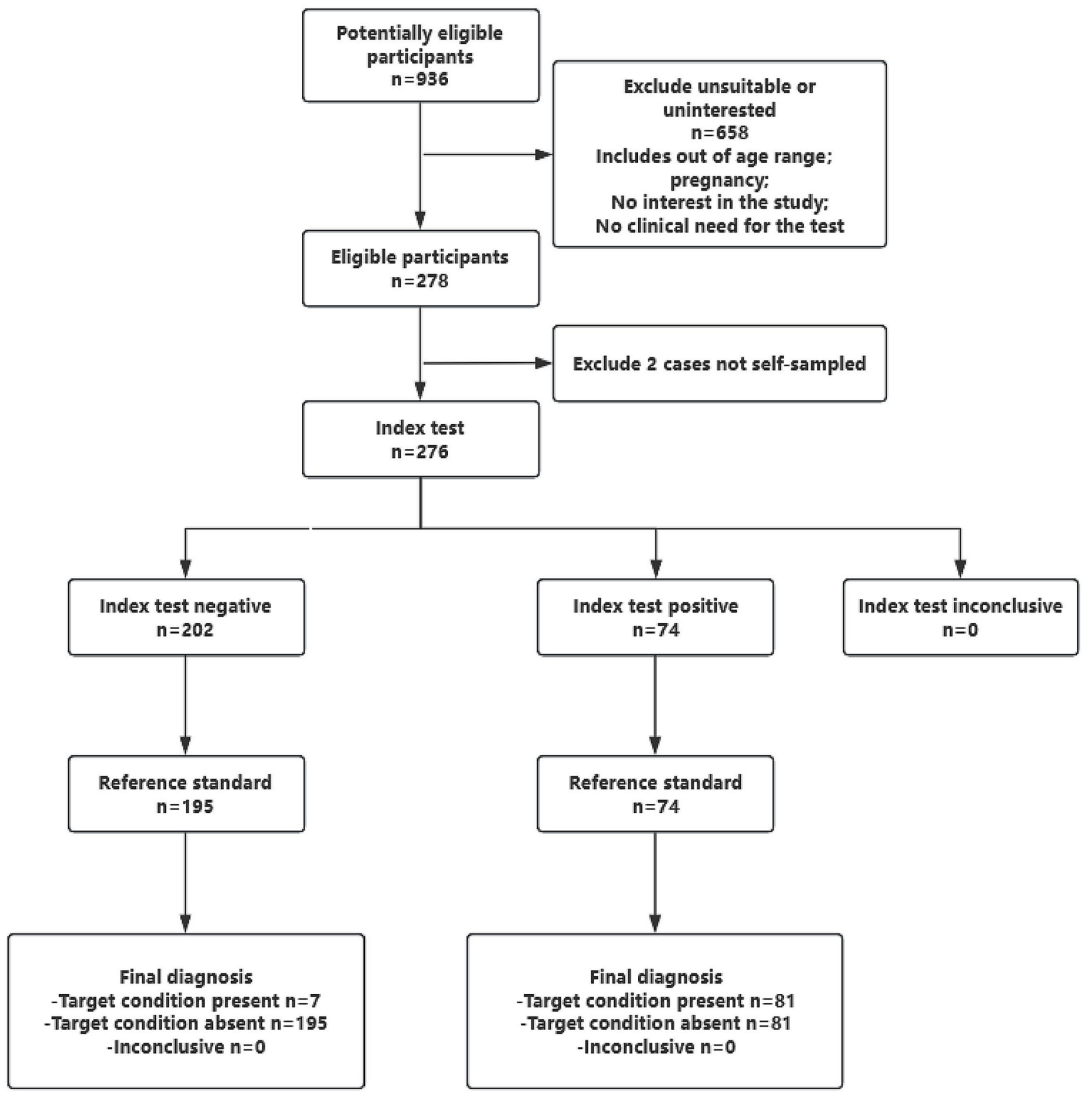
Study flow diagram. This diagram illustrates the participant enrollment process, sample collection protocol, and data analysis workflow.

As shown in Figures 2, 936 women attending the gynecology outpatient clinic at Shanghai East Hospital underwent initial screening for eligibility. Of these, 658 were excluded due to unmet inclusion criteria or refusal to participate, resulting in 278 enrolled participants. During the study, two participants were excluded for incomplete self-sampling procedures. Consequently, 276 cases with complete paired-sample datasets were available for HPV analysis. Valid questionnaires were obtained from 268 participants, while eight completed HPV testing but did not submit their questionnaires. Table 1 summarizes the demographic and clinical characteristics of the cohort. The mean age was 43.6 ± 9.8 years, with the largest proportion (47.8%) belonging to the 31–45-year age group. HPV vaccination coverage was 35.1, and 60.5% of participants reported prior HPV testing. Notably, 21.6% had never undergone any form of cervical cancer screening.

**Table 1:** Basic characteristics of the study subjects (*N* = 268).

Characteristics	Classification	Number	Percentage (%)
Age (years)	0–30	47	17.5
	31–45	128	47.8
	46–60	73	27.2
	≥61	20	7.5
HPV Vaccination Status	Yes	94	35.07
	No	171	63.81
	Not sure what HPV vaccine is	3	1.12
Previous Cervical Cancer Screening *	Pap smear/TCT	120	44.78
	HPV testing	162	60.45
	Clinician examination (VIA/VILI)/Colposcopy	43	16.04
	Not sure if screened	22	8.21
	Never screened	58	21.64
Time Since Last HPV Test	Within 1 year	81	30.22
	Within 3 years	67	25.00
	Within 5 years	19	7.09
	More than 5 years	14	5.22
	Not sure what HPV testing is	17	6.34
	Never tested	70	26.12
Location of Previous HPV Testing	Hospital	157	58.58
	Physical examination center	42	15.67
	Self-sampling at home + mail sample + mobile report	1	0.37
	Not sure what HPV testing is	6	2.24
	Never tested	76	28.36
	Other	2	0.75

*Previous Cervical Cancer Screening is multiple choice, with percentages totalling more than 100 per cent.

### Agreement in HPV detection between sampling methods

As shown in Table 2, the comparison of HPV test results between self-sampling and clinician sampling revealed a positive concordance rate of 91.4% (95% CI: 83.2–96.5%) and a negative concordance rate of 100% (95% CI: 98.2–100%). The overall concordance (P_0_) was 97.5%, with a Kappa coefficient of 0.937 (95% CI: 0.89–0.98), indicating excellent agreement between the two methods. When the analysis was restricted to the 18 high-risk HPV types, the concordance remained high: positive concordance 81.0% (95% CI: 68.0–90.1%), negative concordance 99.5% (95% CI: 97.5–100%), overall concordance 95.6%, and a Kappa coefficient of 0.911 (95% CI: 0.85–0.97).

**Table 2:** Agreement in HPV test results (positive/negative) between self-sampling and clinician sampling (*N* = 276).

Outcome	Positive clinician sampling	LR-HPV only clinician sampling	Negative clinician sampling	Total
Any positive Self-sampling	74	/	0	74
Negative Self-sampling	7	/	195	202
Total	81	/	195	276
HR-HPV only Self-sampling	47	1	0	48
LR-HPV only Self-sampling	4	22	0	26
Negative Self-sampling	7	0	195	202
Total	58	23	195	276

TP, true positive; FP, false positive; FN, false negative; TN, true negative; HR, high risk; LR, low risk.

### Concordance metrics

The following metrics describe the agreement between self- and clinician-sampling for detecting HPV, not for diagnosing cervical precancer. Using clinician sampling as the reference standard, the diagnostic performance of self-sampling is summarized in Table 3. Self-sampling demonstrated a sensitivity of 91.4% (95% CI: 83.2–96.5%), specificity of 100% (95% CI: 98.2–100%), accuracy of 97.5% (95% CI: 94.8–99.0%), positive predictive value of 100% (95% CI: 95.1–100%), and negative predictive value of 96.5% (95% CI: 93.0–98.5%). When the analysis was restricted to the 18 high-risk HPV types, the concordance performance metrics for HR-HPV were: sensitivity 81.0% (95% CI: 68.0–90.1%), specificity 99.5% (95% CI: 97.5–100%), accuracy 95.6% (95% CI: 92.5–97.7%), PPV 97.9% (95% CI: 88.9–100%), and NPV 95.2% (95% CI: 91.5–97.6%).

**Table 3:** Concordance metrics for self-sampling compared to clinician sampling.

Outcomes	Indicator	Formula	Value (95% CI)
Any	Sensitivity	TP/(TP + FN)	91.4% (83.2–96.5%)
	Specificity	TN/(TN + FP)	100% (98.2–100%)
	Accuracy	(TP + TN)/Total	97.5% (94.8–99.0%)
	PPV	TP/(TP + FP)	100% (95.0–100%)
	NPV	TN/(TN + FN)	96.5% (93.2–98.5%)
HR-HPV	Sensitivity	TP/(TP + FN)	81.0% (68.0–90.1%)
	Specificity	TN/(TN + FP)	99.5% (97.5–100%)
	Accuracy	(TP + TN)/Total	95.6% (92.5–97.7%)
	PPV	TP/(TP + FP)	97.9% (88.9–100%)
	NPV	TN/(TN + FN)	95.2% (91.5–97.6%)

Among the seven false-negative results from self-sampling, further analysis revealed that the missed infections primarily involved HPV type 58 (three cases), HPV type 16 (two cases), and HPV type 52 (two cases). This pattern suggests potential variability in detection sensitivity across HPV subtypes with self-sampling, or possible contributing factors such as non-adherence to sampling protocols, inadequate sampling depth or volume, or variability in lesion location.

To preliminarily assess the potential clinical significance of the false-negative self-samples, we reviewed available contemporaneous ThinPrep cytology test (TCT) results from the participants’ clinical records. Among the seven cases where self-sampling failed to detect HPV that were detected by clinician sampling, the cytology findings indicated all of cases of ‘Negative for Intraepithelial Lesion or Malignancy (NILM)’. No High-grade Squamous Intraepithelial Lesion (HSIL) cytology was reported in these false-negative cases. However, the correlation between cytology and HPV status is imperfect, and the definitive assessment of clinical safety requires future studies with colposcopy and histopathology follow-up for all HPV-positive individuals.

### Sampling method preference analysis

Among the 268 questionnaire respondents, 41.4% preferred cervical self-sampling, 32.1% preferred clinician sampling, and 26.5% indicated no preference. However, when cost was considered (approximately CNY 200 for self-sampling vs. approximately CNY 300 for clinician sampling, plus associated time costs), the preference for self-sampling increased significantly to 56.7%, while preference for clinician sampling decreased to 19.8%; 23.5% remained indifferent. This significant shift (a 15.3-percentage-point increase) suggests that financial considerations substantially influence patients’ choice of sampling method.

The primary reasons cited for preferring self-sampling were: the comfort and reduced embarrassment of sampling at home (59.2%), the ability to control sampling pressure to reduce pain (57.2%), privacy protection (56.6%), and time flexibility and convenience (41.5%). These proportions were similar, indicating that multiple interrelated factors collectively influence the preference for self-sampling. Conversely, the main reasons for choosing clinician sampling were perceived professionalism and reliability (73.6%) and higher confidence in hospital test report accuracy (56.6%). Only 9.4% of participants chose clinician sampling because they were hesitant to perform the procedure themselves.

### Sampling comfort and pain analysis

Regarding comfort, 56.0% of respondents considered self-sampling more comfortable, while only 23.1% considered clinician sampling more comfortable; 20.9% found both methods equally comfortable. On a 10-point comfort scale (10 = most satisfied), 76.9% of respondents rated the self-sampling device as “very satisfactory” (score of 10), compared to only 49.3% for clinician sampling, indicating a significant advantage in perceived product comfort for self-sampling (*χ*^2^ = 38.44, *p* < 0.001).

**Figure 3:**
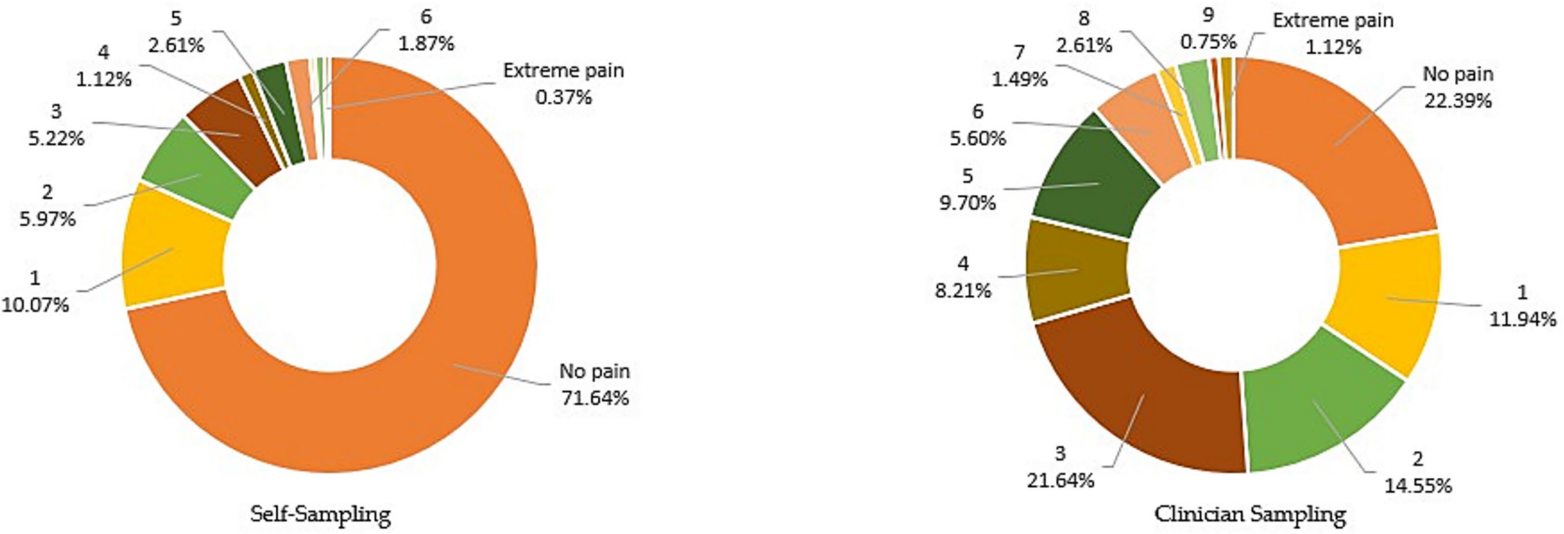
Pain assessment comparison between self-sampling and clinician-sampling methods.

In terms of pain, 67.9% of respondents reported self-sampling as less painful, while only 11.9% considered clinician sampling less painful; 20.2% perceived no significant difference. Specifically, 71.6% of users rated self-sampling as pain-free (score of 0), 21.3% reported mild pain (scores 1–3), and only 7.1% reported moderate or greater pain (scores 4–10). In contrast, only 22.4% rated clinician sampling as pain-free, while 48.1% reported mild pain and 29.5% reported moderate or greater pain. The substantial difference in pain-free rates (71.6% vs. 22.4%) further confirms the clear advantage of self-sampling in minimizing patient discomfort (Figure 3).

### Analysis of factors influencing sampling mode preference

Chi-square analysis revealed that sampling method preference was significantly associated with age, HPV vaccination status, prior screening history, time since last HPV test, subjective comfort during sampling, and self-sampling product comfort/pain scores (*p* < 0.05; Table 4). This indicates that preference is shaped by a combination of individual characteristics, healthcare behaviors, and procedural experiences.

**Table 4:** Analysis of factors influencing preferences in sampling methods.

Variable	Self-sampling (%)	Clinician sampling (%)	Either/No preference (%)	*χ* ^2^	*p*	Cramér’s V
Age group				29.99	0.00001	0.264
0–30	34.0	29.8	36.2			
31–45	31.3	38.3	30.5			
46–60	54.8	27.4	17.8			
≥61	75.0	15.0	10.0			
Vaccination status				14.29	0.0064	0.163
Vaccinated	26.6	39.4	34.0			
Not vaccinated	49.1	28.7	22.2			
Screening history				32.61	<0.0001	0.248
Screened	32.6	39.5	27.9			
Unsure	35.0	35.0	30.0			
Never screened	72.4	6.9	20.7			
Time since last HPV test				29.58	0.0010	0.235
Within 1 year	21.0	50.6	28.4			
More than 1 year	44.0	30.0	26.0			
Never tested	65.7	14.3	20.0			
Comfort evaluation				194.93	<0.00001	0.616
Self-sampling more comfortable	70.7	12.7	16.6			
Clinician sampling more comfortable	3.2	88.7	8.1			
About the same	5.4	21.4	73.2			
Self-sampling pain rating				59.28	<0.00001	0.352
No pain (0)	49.5	26.6	24.0			
Slight pain (1–3)	22.8	42.1	35.1			
Moderate-to-severe pain (≥4)	10.5	57.9	31.6			

Further multivariable logistic regression analysis identified three independent predictors of a preference for self-sampling: higher comfort score (OR = 9.78, 95% CI: 5.12–18.67), age ≥46 years (OR = 2.81, 95% CI: 1.43–5.52), and no prior screening history (OR = 3.21, 95% CI: 1.58–6.53). Notably, HPV vaccination history (OR = 0.67, 95% CI: 0.37–1.22) was not independently associated with self-sampling preference.

### Evaluation of self-sampling practices

The survey revealed that 91.0% of participants performed cervical self-sampling successfully and independently without challenges. Among the 9.0% who reported difficulties, operational uncertainties were predominant: confusion about detaching the swab tip or preserving the sample (58.3%), uncertainty about sampling adequacy or removal timing (41.7%), ambiguity regarding insertion depth markers (16.7%), and questions about the rotational technique during sampling (16.7%). These findings highlight critical gaps in user comprehension, particularly regarding technical execution and procedural clarity, which directly inform the need for targeted improvements in instructional materials and competency-based training protocols.

## Discussion

This study demonstrates that the novel cervical self-sampling device achieves high concordance for HPV genotyping, with near-perfect concordance to clinician-collected samples (*κ* = 0.937, 95% CI: 0.89–0.98). Its sensitivity was 91.4% (83.2–96.5%), specificity 100% (98.2–100%), and overall accuracy 97.5% (94.8–99.0%). No statistically significant difference was observed in high-risk HPV detection rates between the two methods, supporting self-sampling as a reliable alternative in screening programs.

The pattern of false negatives, primarily involving HPV types 58, 16, and 52, warrants further investigation. Several non-mutually exclusive factors could contribute to this. First, it may be related to viral load; samples with lower viral loads might be more susceptible to sampling variability. Second, anatomical factors could play a role. The device’s design, while ergonomic, may have variations in its ability to consistently collect cells from all cervical transformation zones, potentially missing focal lesions. Third, user-dependent factors, such as subtle variations in insertion depth, rotation technique, or sampling pressure, might affect the adequacy of the specimen for these specific genotypes. Interestingly, HPV 58, 52, and 16 are among the most prevalent high-risk types in China. Future studies should combine self-sampling with concurrent viral load quantification and detailed colposcopic examination to determine if there is a genotype-specific or lesion-location-specific sampling efficacy that needs to be addressed through iterative device design or user instruction refinement.

The acceptability analysis revealed a tiered preference for self-sampling, with 41.4% of participants expressing an immediate preference that increased to 56.7% when cost considerations were introduced. The significant increase in preference for self-sampling (from 41.4 to 56.7%) when a lower price point was introduced suggests that women in this developed urban setting are highly sensitive to the total cost of screening, which encompasses more than the test itself. This ‘total cost’ includes hidden or opportunity costs such as clinic registration fees, transportation expenses, and, importantly, the time cost associated with taking time off work, arranging childcare, and waiting at the healthcare facility. Self-sampling eliminates or reduces many of these indirect costs and logistical burdens. Therefore, the price differential presented in the questionnaire may have acted as a tangible proxy for these broader economic and convenience trade-offs, making the value proposition of self-sampling more apparent. Three patient-centered factors emerged as key drivers for choosing self-sampling: comfort (59.2%), privacy protection (56.6%), and reduced pain perception (57.2%). These findings align with a large-scale validation study (*N* = 8,136) conducted in remote regions of China (25), where self-sampling acceptance reached 62.4%, with comparable motivators including convenience (32.7%), privacy (21.8%), and pain reduction (21.2%). Proportional differences between the studies are attributable to distinct questionnaire designs (single- versus multiple-choice formats).

The highest acceptance rate (72.4%) was observed among cervical cancer screening–naïve women, underscoring self-sampling’s potential to bridge screening gaps in historically underserved populations. This evidence base supports the targeted deployment of direct-mail self-sampling kits to priority groups, including due or overdue screeners and non-adherent populations, where this modality has demonstrated enhanced screening efficacy (26). Furthermore, self-sampling addresses critical healthcare challenges by facilitating HPV testing implementation in resource-limited settings lacking routine screening infrastructure while simultaneously providing a viable alternative for maintaining cervical cancer screening continuity during public health emergencies such as the COVID-19 pandemic (27, 28).

While self-sampling excels at improving initial screening coverage, ensuring successful completion of the clinical management pathway for screen-positive women is paramount for program effectiveness. A large Dutch population-based study (*n* = 840,428) revealed a critical implementation challenge: HR-HPV–positive women identified through self-sampling faced significantly higher risks of triage noncompliance, colposcopy attrition, and loss to follow-up compared with those whose samples were clinician-collected (29). This evidence highlights that the successful integration of self-sampling into national programs requires a tripartite strategy that extends beyond the test itself: (1) establishing rigorous, patient-centric tracking and recall systems for self-sampling–positive individuals; (2) implementing enhanced health literacy interventions that clearly explain the meaning of a positive HPV result and the necessity of follow-up; and (3) developing and validating streamlined clinical management protocols that minimize the number of required clinical visits. Such supportive infrastructure is essential to mitigate downstream attrition risks and ensure that the coverage gains achieved by self-sampling translate into true reductions in cervical cancer incidence and mortality.

The prospective matched-pair design allowed direct intra-individual comparison, minimizing inter-subject variability. The study also systematically linked demographic factors—such as older age (≥46 years) and lack of prior screening—to a preference for self-sampling, offering insights for tailored implementation. Furthermore, the device was ergonomically adapted to the cervical anatomy of Chinese women, which may improve sampling adequacy.

Several limitations should be acknowledged. The most important limitation of this study is that it was designed as a concordance study for HPV detection, not as a diagnostic accuracy study for cervical intraepithelial neoplasia. We did not obtain histopathological confirmation (colposcopy/biopsy) for HPV-positive participants. Therefore, the clinical sensitivity and specificity of this self-sampling device for detecting CIN2 + remain unknown and must be established in future longitudinal studies. The moderate sample size (*N* = 276) may constrain subgroup analyses, and recruitment from a single urban hospital limits generalizability to rural populations. While participants performed self-sampling privately, a research assistant was on standby nearby. This supervised clinic environment may have provided an implicit sense of support and opportunity for immediate clarification, potentially leading to better adherence to instructions and higher sampling adequacy than might be achieved in a completely unsupervised home setting. The real-world performance and user experience of purely home-based self-sampling require further investigation. Additionally, cost-related preferences were assessed hypothetically rather than through actual payment decisions, which could inflate stated willingness. Longitudinal data on long-term outcomes such as CIN2 + detection and cancer incidence are also lacking.

Self-sampling offers a patient-centered complement to clinician-based screening, particularly for expanding coverage among under-screened groups. The significant shift in preference when cost was considered—even in an economically developed setting—suggests that pricing and insurance reimbursement policies could substantially influence uptake. Integrating self-sampling into existing healthcare pathways—through hospital-based distribution, community training, and digital support—could enhance accessibility and adherence. Future research should prioritize: (1) large-scale multicenter validation across diverse demographic and geographic groups; (2) Real-world accuracy studies in fully unsupervised home environments; (3) Development of digital aids, such as video-guided tutorials and AI-based quality assessment tools; (4) Longitudinal studies evaluating long-term clinical impact on precancer detection and cancer incidence; and (5) Comprehensive cost–benefit analyses incorporating direct medical costs, time savings, and systemic burdens.

## Conclusion

This study demonstrates that the novel cervical self-sampling device shows high concordance with clinician sampling for HPV detection and good acceptability, positioning it as a promising screening tool whose clinical performance for lesion detection warrants further investigation. Successful implementation will require addressing key operational considerations, including user training, follow-up protocols for positive results, and potential variations in detection sensitivity across HPV genotypes. With continued technological refinement and practical experience, self-sampling is well-positioned to become a valuable component of China’s cervical cancer prevention strategy, enhancing access to early detection.

## Data Availability

The original contributions presented in the study are included in the article/Supplementary material, further inquiries can be directed to the corresponding author/s.
